# Patient’s view on better care

**DOI:** 10.1007/s12020-021-02653-w

**Published:** 2021-02-24

**Authors:** Jette Kristensen, Manuela Brösamle, Bas van den Berg

**Affiliations:** 1 Addison Foreningen i Danmark, Skodstrup, Denmark; 2 AGS-Eltern- und Patienteninitiative e.V. Germany, Kötz, Germany; 3 Dutch Adrenal Patient Society NVACP, Nijkerk, The Netherlands


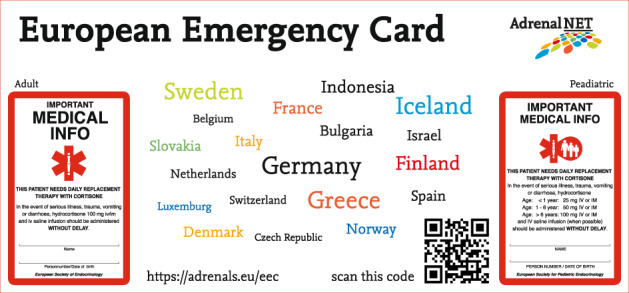


## To the Editor:

Adrenal insufficiency is characterized by insufficient production of glucocorticoids and in case of primary adrenal insufficiency also of mineralocorticoids by the adrenal cortex. Daily corticosteroid replacement therapy is the standard treatment. Other adrenal diseases that can result in adrenal insufficiency are for instance ACTH independent Cushing’s syndrome (after treatment) and congenital adrenal hyperplasia.

Care for patients with adrenal disease has improved enormously in the past 10 years. A great deal of research has been carried out. There has been intensive collaboration between patient associations and healthcare professionals to ensure better care and to ensure that patients and their carers/parents are better informed about the disorder.

Despite a clearer understanding of the importance of more precise replacement therapy, patients are still reporting a reduced quality of life. Their ‘complaints’ in this context relate not only to physical symptoms such as fatigue or muscle and joint pain, but also to psychosocial problems [1] especially in patients with comorbidity. Psychological wellbeing has increasingly been the subject of research during the last decade.

The key factors for improving quality of life are early diagnosis, improved dosage of medication (3–4 times daily) and increased self-management and knowledge about the disorder for both patients and their immediate circle of carers. New medications like slow-release and modified release hydrocortisone preparations and hydrocortisone tablets with lower dosages improve and better reflect the naturally occurring circadian endogenous release of glucocorticoids [2]. At the same time, steps have been taken towards research that will improve the psychological support and the quality of life for patients.

A close collaboration between the patient associations and the healthcare professionals will ensure a better care through education.

In addition, various aids have been developed to give patients and healthcare practitioners a better understanding of the disorder and help them take the necessary actions in times of increased stress. It has become clear that good education of the patient and his/her close relatives and better cooperation between the patient and the healthcare practitioners is essential to reduce morbidity and mortality [3].

About 10 years ago an emergency card was developed in Sweden based on the idea is that patients should carry the card for their own safety so that it can be shown to healthcare providers (GPs, ambulance crews, emergency room staff, etc.) in the event of an emergency. By making it known that the patient suffers from an adrenal disease, a stress medication schedule can be instigated immediately to avoid a life-threatening adrenal crisis.

At the request of the Swedish doctors and with the blessing of the ESE and the ESPE in 2015, AdrenalNET developed this card into what it is now—the standard European Emergency Card. The card is produced in two editions, a paediatric and an adult version, in no <17 countries [4].

Besides the large-scale production of the physical plastic European Emergency Card, individual patients can download a template from www.adrenals.eu and print a card for their own use. This website also offers a selection of information and instruction material in several languages for patients.

The collaboration between German, Scandinavian, and Dutch parties has proven to be beneficial to improve the education, the knowledge, and thus the position of individual patients and their carers/parents. Particularly in times of acute stress and a risk of adrenal crisis, it is crucial that a patient and his immediate circle know what to do and how to act even when no medical professionals are around.

Unfortunately, the education of patients and their partners is still not optimal in all European countries.

The patient representatives within Endo-ERN-MTG1 (Adrenal) are eager to work with the medical specialists in all the member states, so that the basis of patient care throughout the European Union is expanded from ‘diagnose’ and ‘treat’ to include two new components: ‘educate’ and ‘equip’. The autonomy of the patient must be increased, and this shall lead to an improved quality of life for patients with an adrenal disorder.

Furthermore, the patient representatives urge the healthcare systems throughout Europe to include psychosocial support as part of the patient care as a standard resource.

By improving care for patients with an adrenal disorder in this way, rolling the concept out all over Europe, the future will see far fewer patients suffering from an adrenal crisis and many more patients enjoying a better quality of life.
